# ERK Pathway in Activated, Myofibroblast-Like, Hepatic Stellate Cells: A Critical Signaling Crossroad Sustaining Liver Fibrosis

**DOI:** 10.3390/ijms20112700

**Published:** 2019-06-01

**Authors:** Beatrice Foglia, Stefania Cannito, Claudia Bocca, Maurizio Parola, Erica Novo

**Affiliations:** Department Clinical and Biological Sciences, Unit of Experimental Medicine and Clinical Pathology, University of Torino, Corso Raffaello 30, 10125 Torino, Italy; bfoglia@unito.it (B.F.); stefania.cannito@unito.it (S.C.); claudia.bocca@unito.it (C.B.); erica.novo@unito.it (E.N.)

**Keywords:** ERK pathway, hepatic stellate cells, liver myofibroblasts, liver fibrosis, chronic liver diseases, antifibrotic strategies

## Abstract

Fibrogenic progression of chronic liver disease, whatever the etiology, is characterized by persistent chronic parenchymal injury, chronic activation of inflammatory response, and sustained activation of liver fibrogenesis, and of pathological wound healing response. A critical role in liver fibrogenesis is played by hepatic myofibroblasts (MFs), a heterogeneous population of α smooth-muscle actin—positive cells that originate from various precursor cells through a process of activation and transdifferentiation. In this review, we focus the attention on the role of extracellular signal-regulated kinase (ERK) signaling pathway as a critical one in modulating selected profibrogenic phenotypic responses operated by liver MFs. We will also analyze major therapeutic antifibrotic strategies developed in the last two decades in preclinical studies, some translated to clinical conditions, designed to interfere directly or indirectly with the Ras/Raf/MEK/ERK signaling pathway in activated hepatic MFs, but that also significantly increased our knowledge on the biology and pathobiology of these fascinating profibrogenic cells.

## 1. Introduction: Fibrogenic Progression of Chronic Liver Diseases

Progression of chronic liver diseases (CLD), whatever the etiology, is typically characterized by an interrelated vicious circle involving persistent chronic parenchymal injury, chronic activation of inflammatory response, and sustained activation of liver fibrogenesis, and of pathological wound healing response. Liver fibrogenesis is a dynamic, highly integrated molecular, cellular, and tissue process that can result in the progressive excess accumulation of extracellular matrix (ECM) components (i.e., liver fibrosis). Literature of the last two decades unequivocally indicates that the fibrogenic progression is primarily sustained by the activation of a heterogeneous population of proliferative, migratory, and pro-fibrogenic cells defined as hepatic myofibroblasts (MFs) that are also involved in the modulation of inflammatory/immune response as well as of angiogenesis [1,2,3,4,5,6,7].

Liver fibrogenesis and fibrosis then represent key features of the progression of virtually any form of CLD, eventually leading to liver cirrhosis and organ failure, with progression being intimately linked also to pathological angiogenesis [8,9]. Liver cirrhosis is an advanced stage of CLD characterized by a deranged organ structure resulting from the formation of regenerative parenchymal nodules surrounded by fibrotic septa and by relevant changes in organ vascular architecture. These structural and vascular changes eventually lead to the development of portal hypertension and related complications seen in cirrhotic patients (variceal bleeding, hepatic encephalopathy, ascites, hepatorenal syndrome, etc.) [10]. Liver cirrhosis at present is the main indication for liver transplantation in Europe and the United States of America (USA). Fibrogenic progression of CLD also exposes patients to a significant risk to develop hepatocellular carcinoma (HCC) that accounts for approximately 75–80% of primary liver malignancies, representing the fifth most common solid malignant tumor and the third leading cause of cancer-related death worldwide [11,12].

The fibrogenic progression of CLD is usually a longstanding process (cirrhosis and related complications develop, on average, after at least 15–20 years of chronic parenchymal injury) and has a major impact on public health. Recent epidemiological studies have estimated that more than 800 million individuals worldwide are affected by a form of CLD, with an impressive mortality rate of approximately 2 million deaths per year [13,14]. Although the estimated worldwide incidence and prevalence of CLD significantly varies also in relation to the geographic area and several critical factors (including sex, socio-economic status, ethnic group, etc.), the most relevant etiologies leading to CLD can be summarized as follows [14,15,16,17]:

- chronic infection by hepatothropic viruses like hepatitis C virus (HCV), globally distributed, and hepatitis B virus (HBV) being predominant in Asia;
- non-alcoholic fatty liver disease (NAFLD), an obesity and diabetes type II-related CLD whose incidence and prevalence is dramatically growing worldwide, particularly in western countries;
- excess ethanol consumption, responsible for alcoholic liver disease (ALD), relevant in western countries;
- autoimmune-mediated form of CLD, including either conditions affecting the biliary tree such as primary biliary cholangitis (PBC) and primary sclerosing cholangitis (PSC), or autoimmune hepatitis (AIH);
- a number of more rare hereditary diseases including Wilson’s disease (WD), α1-anti-trypsin (α1-AT) deficiency, and the different genetic variants of hemochromatosis.

**Figure 1:**
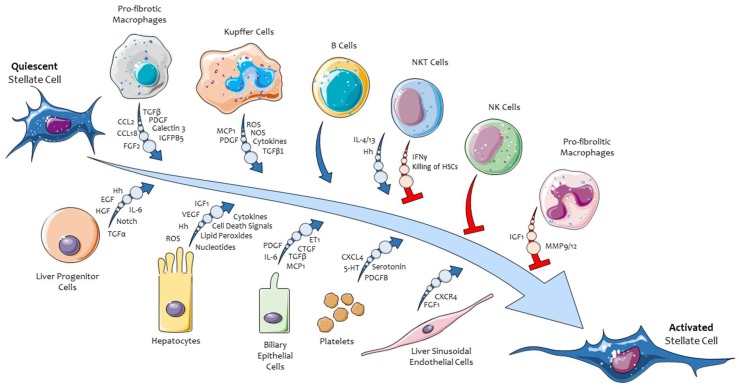
The process of activation/trans-differentiation of hepatic stellate cells into activated, myofibroblast-like cells (HSC/MFs) as modulated positively (blue arrows) or negatively by mediators released by different hepatic cell populations involved in chronic liver injury.

In order to introduce the pro-fibrogenic role of hepatic MFs and, in particular, of hepatic stellate cells (HSC), it should be recalled that CLD progression is critically sustained by chronic inflammatory response. Chronic hepatitis involves mainly the activation of either resident macrophages (i.e., Kupffer cells) and macrophages derived from monocytes recruited from peripheral blood, as well as other cells of innate and adaptive immunity. The activation of innate and adaptive immune cells, occurring through the release of several soluble peptide mediators (cytokines, growth factors, chemokines) and reactive oxygen species (ROS) generation is critical in initiating and perpetuating the activation of pro-fibrogenic hepatic MFs [7,18,19]. In turn, hepatic MFs contribute to CLD perpetuation and progression not only by synthetizing and releasing ECM components but also by actively releasing cytokines, chemokines, peptide growth factors, and other mediators. Activated MFs, inflammatory cells, and other liver cell populations then establish a “pro-fibrogenic environment” which is critical for CLD progression and is able to negatively affect proliferation of parenchymal liver cells (i.e., hepatocytes) [3,4,5,8,18,20]. Figure 1 is offering a synthetic summary of such a complex chronic injury environment by highlighting most relevant mediators released by the different cell populations involved in CLD progression. A more detailed analysis of events, mediators cells, and mechanisms involved can be found in recent authoritative and comprehensive reviews [1,2,3,4,5,6,7,8,18,19,20,21,22]. In this review we would like to focus the attention on the role of extracellular signal-regulated kinase (ERK) signaling pathway as a critical one in modulating selected phenotypic responses of liver myofibroblasts. These critical profibrogenic cells mainly originate from hepatic stellate cells (HSC) and from a limited number of other cellular sources [21,22,23,24,25,26,27]. We will then dedicate a section to therapeutic strategies developed in preclinical studies and translated to clinical conditions designed to interfere with the Ras/Raf/MEK/ERK signaling pathway in activated hepatic MFs.

## 2. ERK Signaling Pathway: A Crossroad Conveying Multiple Signals and Modulating Different Cellular Responses

**Figure 2:**
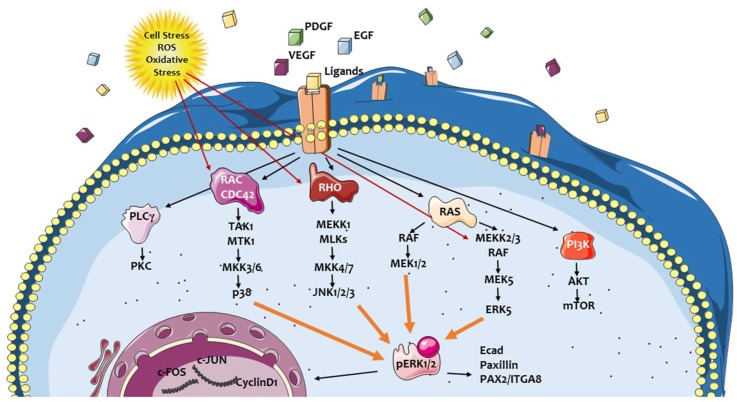
Mitogen-activated protein kinase (MAPK) cascades in activated, myofibroblast-like hepatic stellate cells (HSC/MFs). Pathways activated by major extracellular peptide ligands active on HSC/MFs or following oxidative stress and reactive oxygen species (ROS) generation.

In a typical hepatic pro-fibrogenic environment, which is common to any form of chronic liver injury, the mitogen-activated protein kinase (MAPK) cascades are definitively involved. As it is well known, MAPK cascades are evolutionary conserved, intracellular signal transduction pathways that are involved in the response to numerous extracellular stimuli. MAPK then control or modulate several critical cellular processes including growth, cell proliferation, differentiation, motility, the response to different cellular stressors, as well as survival and apoptosis [28,29,30]. Any MAPK cascade usually consists of at least three core kinases, defined as MAP3K, MAPKK, and MAPK, as well as of additional components operating either upstream (such as MAP4K) or downstream (MAPKAPK). Once activated, the signal is propagated through sequential phosphorylation and activation of sequential kinases. This, in turn, leads to the phosphorylation of hundreds of target regulatory proteins identified in the cytoplasm, mitochondria, endoplasmic reticulum, and Golgi apparatus, as well as in the nucleus [28,29,30,31,32,33]. Transmission of signals to the nucleus is operated mostly by a stimulated physical translocation of MAPK cascade components. This nuclear translocation is critical since the most common role of these pathways is represented by induction and regulation of gene expression through the modulation of several transcription factors, but also transcription suppressors and chromatin remodeling proteins [31,32,33]. At present, at least four distinct mammalian MAPK cascades have been identified, including extracellular signal-regulated kinase 1 and 2 (ERK1/2), c-Jun N-terminal kinase (JNK), p38 MAPK, and ERK5. According to the subject of the present review, Ras/Raf/MEK/ERK signaling cascade is a key signaling pathway which integrates extracellular signals from cell surface receptors to gene expression and regulation of multiple cellular proteins. ERK cascade plays a crucial role in cell proliferation, differentiation, adhesion, migration, and survival. In particular, ERK cascade is critical in supporting liver fibrogenesis by acting as a major signaling pathway involved in defined phenotypic responses of hepatic MFs (Figure 2) [1,2,3,5,7].

From an historical point, the ERK1/2 cascade has been the first MAPK pathway elucidated [34] and consequently is widely considered as the prototype of MAPK cascades, known to play a central role in transmitting signals from several extracellular agents operating via different receptors. In most cases, the involvement of these receptors conveys, through different mechanisms, the activation (mainly at the plasma membrane level) to the small GTPase Ras. Ras, in turn, can recruit to the plasma membrane, the so-called MAP3K tier of the cascade, in particular Raf-1 and B-Raf, leading eventually to their activation. Activation of Rafs is operated likely through homo- or hetero-dimerization and phosphorylation by different kinases including possibly, at least in some conditions, to protein kinase C (PKC) or MLK3. The activating signal is then transmitted to the conventional MAPKKs, MEK1, and MEK2 (MEK1/2) through phosphorylation of two critical serine residues in their activation loop [28,29,30]. Once activated, MEK1/2 phosphorylate the regulatory threonine (Thr) and tyrosine (Tyr) residues in the Thr-Glu-Tyr domain of the ERK1 and ERK2 (ERK1/2) activation loop. Finally, the signal is then transmitted to the MAPKAPK components (RSKs, MSKs, and MNKs) and/or several other substrates distributed in the cytoplasm or subcellular organelles [28,29,30,31,32,33,34]. ERK1/2 can also directly bind to DNA sequences acting as transcriptional repressors of several cytokine-induced genes, particularly those induced by interferon-γ [35]. The phosphorylation of hundreds of substrates results in the induction of several ERK1/2-dependent processes. These include mainly cell proliferation and differentiation but also morphological changes or determination, cell plasticity, and survival, as well as in conditions of cellular injury, stress response, and apoptosis [36].

According to literature data, the most relevant nuclear functions of ERK1/2 cascade can be summarized (as reviewed some years ago [31]) as follows:

- ERK1/2 cascade can regulate immediate early-genes following extracellular stimulation (i.e., by growth factors) through the regulation of a number of transcription factors; a classic example is represented by activation of Elk1, a nuclear ETS domain transcription factor, which is rapidly phosphorylated following direct binding of the CD/CRS domain of ERK1/2 with the D-domain of Elk1; activation of Elk1 leads to the induction of c-Fos, which is critical for proper progression of cell proliferation and differentiation;
- ERK1/2 can regulate transcriptional suppression, as is the case of ETS2 repressor factor Erf1, which is known, in its dephosphorylated and nuclear located form, to suppress transcription in resting and/or serum starved cells; following activation by a mitogen, Erf1 is phosphorylated by ERK1/2 and exported from the nucleus then alleviating its role in suppressing transcription. Prevention of Erf1 phosphorylation has been reported also to arrest fibroblast proliferation in the G0/G1 phase of the cell cycle. Additionally, ERK1/2 suppressing function can result also by the direct interaction (particularly of ERK2) with DNA, through specific binding to the DNA sequence C/CAAAG/C independently on its own catalytic activity [35];
- ERK1/2 cascade is involved in the chromatin remodeling which is relevant, following proper stimulation, to allow proteins (mainly transcription factors) to access and bind to their specific DNA sequences. ERK1/2 cascade has a role in histone deacetylation, phosphorylation of specific chromatin-rearranging protein histones H3 and H4, or by non-conventional influence on PolyADP ribose-polymerase 1 (PARP1);
- ERK1/2 cascade can finally regulate the general nuclear import machinery by interactions with the so-called nuclear pore complexes that control nuclear-cytoplasmic exchange of different molecules.

## 3. Hepatic Myofibroblasts, Their Pro-Fibrogenic Phenotypic Responses, and the Role of ERK Signaling

A critical role in liver fibrogenesis is played by hepatic MFs, a heterogenous population of α smooth-muscle actin (α-SMA)-positive cells. These cells originate from various precursor cells (either mesenchymal or not) through a process of activation and trans-differentiation. This process has been originally characterized for MFs derived from hepatic stellate cells (HSC), a peculiar population of liver cells residing in the space of Disse [21,22]. HSC represents the most relevant hepatic MFs precursor cells in either clinical or experimental conditions, and these cells are sometimes indicated as HSC-MFs. A significant contribution to MFs population comes also from portal fibroblasts (i.e., portal MFs), particularly relevant in the condition of biliary-like fibrosis (i.e., PSC and PBC). Bone marrow-derived cells like mesenchymal stem cells (MSC) and fibrocytes [1,2,3,7,21,22,23,24] likely give raise to a limited number of MFs. The hypothesis that hepatic MFs may originate from hepatocytes or cholangiocytes, through a process of epithelial to mesenchymal transition (EMT) is at present highly debated and controversial. The prevailing view suggests that EMT is likely of minor relevance in liver fibrogenesis [7,24,25,26]. Finally, a few MF may originate from liver mesothelial cells of the Glisson’s capsule through a process of mesothelial mesenchymal transition (MMT) [27].

Persistently activated pro-fibrogenic liver MFs, whatever their origin, seem to operate a rather common number of critical phenotypic pro-fibrogenic responses by acting as a unique crossroad cell type. During CLD progression, MFs can actively integrate (and respond to) an impressive scenario of incoming “signals” (i.e., growth factors, cytokines, chemokines, ROS, adipokines, etc). These signals are released by liver cell populations (hepatocytes, Kupffer cells, sinusoidal endothelial cells or SEC, cholangiocytes, hepatic progenitor cells or HPC, resident lymphocytes) as well as by cells infiltrating innate and adaptive immune cells recruited into injured liver. In addition, several signals in the fibrogenic scenario are also released by activated hepatic MFs themselves (autocrine/paracrine loop), including transforming growth factor-β1 (TGFβ1), C-C motif chemokine ligand 2 (CCL2), platelet-derived growth factors (PDGF), endothelin-1 (ET-1), and vascular-endothelial growth factor A (VEGF-A) [5,6,7,22]. These concepts are once again summarized in Figure 1.

**Figure 3:**
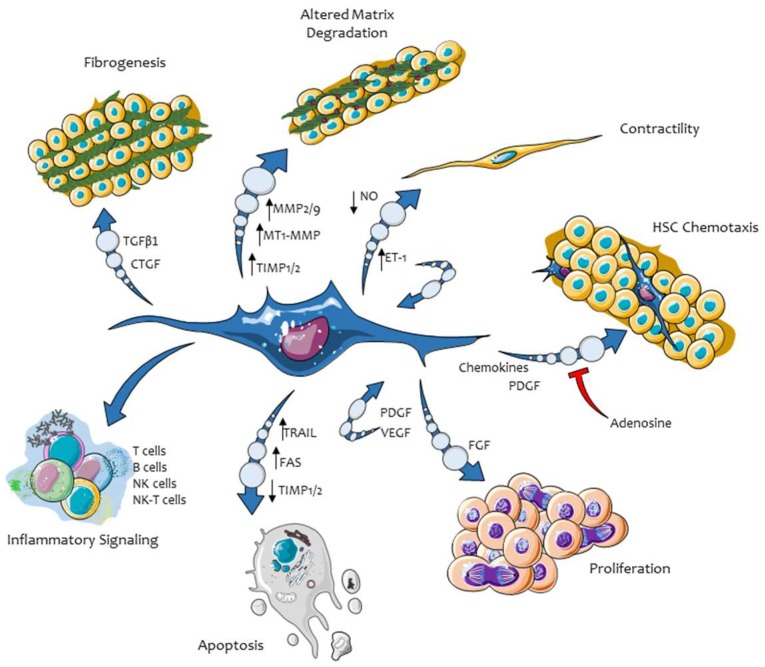
Major phenotypic responses in activated, myofibroblast-like cells (HSC/MFs) as well as, more generally, in hepatic MFs.

The major phenotypic responses elicited by activated hepatic MFs are now well established and most of them are remarkably similar, and sometimes even homologous, to those attributed to MFs in other organs or tissues in the frame of progressing chronic injury. The most relevant and established phenotypic responses operated by liver MFs include: proliferation and survival, synthesis and remodeling of ECM, migration in response to chemoattractants and ROS, pro-inflammatory and immune-modulatory role, as well as a pro-angiogenic role (Figure 3).

### 3.1. Proliferation and Survival of Hepatic MFs

During the progression of CLDs, hepatic MFs are characterized by a high proliferative attitude as a result of the increased availability in the pro-fibrogenic environment of growth factors released by activated neighboring cells and the increased expression of related receptors by MFs themselves. The most potent mitogen for hepatic MFs is without any doubt PDGF, particularly PDGF-BB isoform, with increased expression of the specific receptors PDGF-Rα or PDGF-Rβ being stimulated by TGFβ1. PDGF-Rβ expression has been reported recently to be critical for progression of murine liver fibrosis in vivo and to contribute to the poor prognosis of human cirrhosis [37]. Several other additional mitogens for hepatic MFs have been identified, including transforming growth factor α (TGFα), epidermal growth factor (EGF), connective tissue growth factor (CTGF, renamed as CCN-2, one of the six members of the matricellular and cysteine rich proteins belonging to the CCN family), basic fibroblast growth factor (bFGF), thrombin, endothelin 1 (ET-1), keratinocyte growth factor (also known as FGF7), as well as the adipokine leptin [1,2,3,4,5,6,7,20,21,22]. Most, if not all, of these mitogenic polypeptides for hepatic MFs, by interacting with their cognate receptors, convey their proliferative stimuli mostly through the ERK1/2 cascade and recruitment of adaptive proteins to intracellular domains of the different receptors involved. The most common and established pathway (involving, for example, ligands binding to receptor tyrosine kinases) is the one involving recruitment and activation of the growth factor receptor-bound protein 2 (GRB2). GRB2 in turn recruits the protein Son of Sevenless (SOS, one of the guanine nucleotide exchange factors), finally leading to the activation of Ras and then of the Ras/Raf/MEK/ERK signalling cascade [38,39]. The mitogenic stimuli described for HSC/MFs (and then for hepatic MFs in general), in addition to TGFβ1 and possibly other mediators, are also believed to act as significant survival factors for MFs [1,2,3,4,5,6,7,20,21,22]. This is relevant, since these MFs are also characterized by the ability to survive in the potentially hostile pro-fibrogenic environment of on-going CLDs. Human HSC/MFs, in particular, have been shown to survive to the induction of apoptosis by the most common pro-apoptotic stimuli as well as to ROS, oxidative stress mediators, and hypoxic conditions [40,41].

### 3.2. Synthesis and Remodelling of Extracellular Matrix (ECM) Components 

CLD progression is typically characterized by an increased synthesis and deposition of ECM components by persistently activated hepatic MFs, including fibrillary collagens type I and III, as well as α-SMA, laminin, and fibronectin. The most potent mediator involved in sustaining this phenotypic response is by far TGF-β1, released either by activated macrophages or, in an autocrine/paracrine loop, by hepatic MFs. Increased synthesis and deposition of ECM is associated in activated MFs to a dysregulation of those genes that are classically involved in the remodelling of ECM. This dysregulation is emphasized by the concomitant inefficient removal of excess fibrillary collagen by metalloproteases (MMPs) and the increased expression of tissue inhibitors of MMPs (TIMPs) [1,2,3,4,5,6,7,20,21,22]. Typical TGF-β1-dependent target genes that are activated through the canonical pathway (involving TGFβ receptor type I and II containing serine/threonine kinase domains, phosphorylation of SMAD2 and SMAD3, interaction of SMAD2/3 with SMAD4 to form a transcriptional complex) are represented by type I collagen and CTGF. The canonical pathway, in addition to increased synthesis and remodelling of ECM, is also believed to contribute to activation/trans-differentiation of precursor cells into activated MFs [5,6,7]. However, TGF-β1 has been reported to operate also through non-canonical or SMAD-independent pathways that involve small GTPases (RhoA and Cdc42) leading to the activation of MAPK (ERK1/2, JNK, p38), with ERK1/2 pathway reported to activate type I collagen and CTGF synthesis. Several other mediators can sustain this peculiar response of activated MFs and the list includes at least CTGF, FGF, and leptin, as well as several growth factors, ligand peptides, and signalling pathways [5,6,7]. In addition, literature data also indicates that ROS and then oxidative stress related mediators are believed to be involved, here as well as in other phenotypic responses of MFs. ROS involvement follows activation of NADPH-oxidase isoforms occurring in parallel with the interaction of growth factors, cytokines, and other active peptides with their respective receptors. This is of interest since (see later in this review) ROS can activate MAPK cascades in MFs. Moreover, aldehydic products like acetaldehyde (i.e., largely produced during ethanol metabolism) or 4-hydroxy-nonenal (HNE), a relevant aldehydic end-product of lipid peroxidation, have been shown to be able to up-regulate expression of ECM components. HNE, in particular, has been reported to up-regulate procollagen type I and TIMP-1 expression [42,43], although these effects were shown to be independent on Ras/Raf/MEK/ERK pathway but rather on the ability of HNE to selectively interact with, and activate, JNK isoforms [43,44].

### 3.3. Migration in Response to Chemoattractants and Reactive Oxygen Species (ROS)

Activated MFs are able to migrate in response to several polypeptide chemoattractants released by activated macrophages and MFs themselves, as well as from other surrounding cells (including hepatocytes, endothelial cells, platelets, and other immune cells) or trapped in the ECM. The migratory response is relevant since activated MFs can align to fibrotic septa in the progressing CLD. The list of mitogens active on hepatic MFs is quite impressive, but the most potent chemoattractant is once again PDGF. Other potent chemoattractants effective on HSC/MFs are CCL2, Angiotensin II, and VEGF-A [1,2,3,4,5,6,7]. As shown in other conditions of organ fibrosis, MFs may be also attracted by additional chemokines of the C-C subfamily (CCL3, CCL4, CCL11, CCL20, CCL21, and CCL22) whose involvement in fibrosis is known to be regulated by IL-4 and IL-13 levels [45]. Of interest, chemotaxis for human HSC/MFs exerted by PDGF, Angiotensin II, CCL2, and VEGF-A has been shown to require the involvement of ligand/receptor-related and NADPH-oxidase-dependent increase in intracellular levels of ROS that, in turn, can lead specifically to the activation of ERK1/2 and JNK1/2 signalling pathways [46]. Any significant increase in intracellular ROS levels in activated MFs can result in enhanced migration of these cells, as also shown in pro-fibrogenic cells exposed to hypoxic conditions (i.e., conditions in which an increased release of mitochondrial ROS has been described) [47].

### 3.4. Pro-Inflammatory and Immune-Modulatory Role

Hepatic MFs, particularly HSC/MFs, have been shown to express receptors for several cytokines and other inflammatory mediators. Moreover, these activated MFs are also able to synthetize and release critical pro-inflammatory mediators, in particular the chemokines CCL2 and CCL21, as well as IL-1β following activation of NLRP3 inflammasome [1,2,3,4,5,6,7,20,21,22]. In the pro-fibrogenic scenario of chronic liver injury, this is relevant, since activated MFs can then actively contribute either to perpetuate inflammatory response as well as to regulate and/or modulate interactions with cells of innate and adaptive immunity.

### 3.5. Proangiogenic Role

Extensive literature data indicates that hepatic MFs, and likely HSC/MFs, can contribute to CLD progression by modulating pathological angiogenesis and vascular remodelling. As a matter of fact, HSC/MFs have been reported to synthetize and release proangiogenic mediators, including VEGF-A, PDGF-BB, Angiopoietin-1 or -2, and hedgehog ligands. At the same time, they also express the correspondent receptors, behaving also as a significant cellular target for all of these proangiogenic stimuli. The pro-angiogenic role of hepatic MFs is critical in the progression of CLDs, since hypoxia-dependent pathological angiogenesis usually precedes or parallels the development of liver fibrosis. This issue has led to the proposal that angiogenesis may drive fibrogenesis and the formation of fibrotic septa [8,9,48].

## 4. Therapeutic Anti-Fibrotic Strategies

In the last two decades, intense basic and translational research activity has been performed to identify targetable mechanisms, signalling pathways, and mediators critical for CLD progression or, theoretically, resolution. The obvious final goal was to elaborate, test, and translate to clinical conditions novel and efficient antifibrotic strategies. As detailed elsewhere [1,2,3,4,5,6,7,22,49], several potential therapeutic targets have been elucidated and a plethora of drugs have been tested in pre-clinical studies. Unfortunately, only a minority of them have been translated to clinical conditions or were actually tested in clinical trials (mainly of Phase II and III). In the next sections, we will briefly recall general therapeutic strategies to counteract fibrogenic progression of CLDs to next focus the attention on therapeutic approaches affecting the profibrogenic role of liver MFs by interfering with the Ras/Raf/MEK/ERK signalling pathway.

### 4.1. General Concepts on Antifibrotic Strategies and Pathogenic Therapeutic Targets to Affect Chronic Liver Disease (CLD) Progression

The primary target for any therapeutic strategy designed to counteract CLD progression is represented, whenever possible, by the withdrawal of the etiological agent or condition involved in chronic liver injury. This is unequivocally established in clinical practice by the excellent results obtained with the last generation of direct antiviral agents (DAA), very efficient in clearing HBV or HCV infection. Similarly, abstinence from alcohol consumption represents a plausible option to limit the progression of alcoholic liver disease (ALD) and/or to favour its regression. Apart from these defined conditions, the major antifibrotic strategies that are actually under consideration will be summarized in the next subsections.

### 4.2. Drugs or Strategies Designed to Minimize Parenchymal Liver Injury

When the withdrawal of the etiological agent is not immediately feasible, a plausible option is to try to reduce the impact of chronic liver injury in order to potentially prevent inflammation and fibrogenic progression. Since in almost all clinical and experimental conditions of CLD, a major role is played by ROS and oxidative stress, literature is reporting a huge number of pre-clinical studies employing so-called “hepatoprotective agents”, some of them being true antioxidant molecules, in the attempt to significantly decrease the impact of hepatocyte injury. To this purpose, several molecules have been tested in rodent models of liver fibrosis, including vitamin E, *N*-acetylcysteine, glutathione, S-adenosyl-methionine, curcumin, and resveratrol [50,51]. These molecules, quite efficient in reducing parenchymal injury and fibrosis in murine studies, were essentially ineffective in clinical trials [7,50,51]. More recently, encouraging results were obtained by administering pan-caspase inhibitors like emricasan and PF-03491390 in experimental models [52,53] and in two clinical trials [54,55].

### 4.3. Drugs or Strategies Designed to Target Activated Macrophages

Several studies performed in the last decade have investigated the strategy to specifically target either activated Kupffer cells (KC) and macrophages recruited from peripheral blood or mechanisms and signalling pathways underlying their recruitment/activation. This therapeutic strategy, recently extensively reviewed elsewhere [49,56,57], has been employed in preclinical murine studies and is currently translated into clinical trials. Within the most interesting approaches, one can recall: i) the attempt to block KC activation by reducing bacterial translocation from the gut, ii) the use of a selective inhibitor of the serine/threonine kinase ASK1 and downstream signalling pathways like selonsertib [58] or the use, in experimental and clinical studies, of the dual oral CCR2/CCR5 antagonist cenicriviroc [59,60].

### 4.4. Drugs or Strategies Designed to Target MFs

This therapeutic approach has been the most productive one in terms of pre-clinical and translational studies, with dozens of drugs tested in the attempt to block or attenuate the activation of MFs and then to limit their specific phenotypic responses (the reader can refer to recent and authoritative reviews for more details [1,2,3,4,5,6,7,22,49]). From a general point of view, the most interesting findings, some already translated to clinical conditions, includes the following strategies:

- To interfere with MF-mediated crosslinking of collagens and elastins by the use of the humanized anti-LOXL2 antibody Simtuzumab (G6-6624);
- to interfere with mechanisms resulting in dysregulation of critical molecular pathways in activated HSC or MFs; this approach is by far the most interesting, with several studies dedicated to either blocking pathways elicited by ligand-receptor interactions (we will focus mainly on these approaches that directly or indirectly target Ras/Raf/MEK/ERK pathway), including those elicited by TGFβ1, PDGF, ligand-receptor-induced signalling pathways, HGF, VEGF/VEGFR, Wnt/β-catenin, EGF/EGFR, Hedgehog, endotelins, cannabinoids, adipokines, retinoid, and vitamin D receptors, integrins, and toll-like receptors (TLRs) [5,6,7,22];
- to interfere with nuclear receptor transcription factors expressed by HSC and MFs, including peroxisome proliferator-activated receptor (PPAR)-γ and PPAR-δ, farnesoid X receptor (FXR), liver X receptor (LXR), vitamin D receptor (VDR), nuclear receptor subfamily 4 group A member 1 (NR4A1), and nuclear receptor subfamily 1 group D member 1 (REV-ERBα) [5,22];
- to interfere with transcription factors that positively contribute to HSC and MF activation, including myocardin-related transcription factor A (MRTF-A), sex-determining region Y-box 9 (SOX9), aryl hydrocarbon receptor (AhR), Yes associated protein (YAP), and Gα-interacting vesicle-associated protein (GIV); similarly, to interfere with transcription factors that negatively affect pro-fibrogenic genes and HSC activation like Kruppel-like factors (KLF6 and -2), GATA binding protein 4 (GATA4), NR4A1, and NR4A2 [5,6,7,22];
- to interfere with epigenetic transcriptional dysregulation, particularly with profibrogenic miRNAs either overexpressed in activated HSC (miR-21, miR-27, mirR-125, miR-195, miR-199a, miR-199b, miR-221, and miR-222), and able to sustain MF phenotypic responses, as well as on antifibrotic miRNAs that are down-regulated in activated HSC (miR15b, miR-16, miR-29, miR-122, miR-133b, and miR-200a) [61].

### 4.5. To Promote Fibrosis Resolution

This approach is potentially the most innovative one and resolution of liver fibrosis and, according to literature data, can be theoretically promoted in two ways:

- by inducing selective elimination, reversion, or senescence of MFs; selective killing of activated MFs has been reported in preclinical studies employing gliotoxin, the nuclear factor-κB (NF-κB) inhibitor BAY 11-7082, or the proteasome inhibitors MG-132 and bortezomib as well as by employing the histone-deacetylase inhibitor nilotinib [5,50]; senescence of activated HSC has been obtained using cysteine-rich protein 61 (CCN-Cyr61), curcumin, or OSU03012, a celecoxib derivative deactivation; reversion of MFs to HSC has been observed following withdrawal of etiological agents;
- by increasing ECM degradation, obtained by either using the LOXL2 inhibitor Simtuzumab, an approach effective in preclinical studies but abandoned since it was found ineffective in clinical trials; alternatively, one may transplant bone marrow-derived cells, particularly “resolutive macrophages”, an attempt under evaluation and designed to promote fibrillary ECM degradation and eventually favour regeneration [5,6,7,22].

## 5. Therapeutic Antifibrotic Strategies Designed to Affect Phenotypic Responses of Hepatic MFs that Operate by Involving Ras/Raf/MEK/ERK Signalling Pathway

One of the most productive antifibrogenic strategy has been represented by the attempt to interfere directly with those mechanisms, resulting in dysregulation of critical molecular pathways in activated HSC or MFs. Preclinical and translational studies have provided an impressive amount of data by employing multiple approaches, and the interested reader can refer to exhaustive recent reviews on this topic [5,6,7,22,49]. According to the subject of the present review, we will focus only on a selection of those anti-fibrogenic strategies that, according to the specific design of intervention, operate either by affecting the Ras/Raf/MEK/ERK cascade or by affecting the pathways upstream to this MAPK cascade (i.e., at present the most productive strategy) in order to interfere with phenotypic responses in MFs. Disappointingly, it should be noted that only a limited number of the therapeutic approaches originated by preclinical studies have been translated into clinical trials, and few studies have reported some benefit in CLD patients. On the other hand, all these studies have provided (and still provide) an astonishing contribution that has allowed hepatologists to understand the biology of these fascinating profibrogenic cells.

### 5.1. Antifibrogenic Drug and Strategies Directly Targeting Ras/Raf/MEK/ERK Cascade in Hepatic MFs

The critical role of ERK pathway was outlined in a pioneer study dedicated to the analysis of the involvement of activated HSC (i.e., HSC/MFs) in liver fibrogenesis; this study was the first to unequivocally show that the ERK pathway was activated in vivo during experimental acute liver injury as well as in activated HSC isolated from the injured rodent livers [62]. The same study also showed that the specific inhibition of ERK pathway, obtained by using the MEK pharmacological inhibitor PD98059, resulted in a reduction of PDGF-BB-induced proliferation and chemotaxis. Some years later, it was shown that both the expression and activity of ERK1/2 were up-regulated in a rodent model of biliary fibrosis and that expression of ERK1/2 positively correlated with the expression of α-SMA and then with the involvement of HSC/MFs [63]. These and other publications (reviewed in [1,2,3,4,5,6,7,22]) opened the way to preclinical studies designed to investigate strategies and drugs able to directly affect ERK cascade. These approaches were intrinsically challenged in their clinical translation by the knowledge that in vivo (i.e., systemic) administration of potent inhibitor of ERK cascade components may lead to severe adverse effects. Nevertheless, these drugs and strategies should be recalled for their historical relevance as well, because they started to elucidate critical concepts and issues related to mediators, mechanisms, and signalling pathways actively involved in the activation/trans-differentiation of hepatic MFs. In the following subsections, we will offer a selection (not exhaustive) of some of the most interesting drugs and approaches used to target ERK pathway in HSC/MFs to counteract liver fibrosis.

#### 5.1.1. Pentoxifylline

Pentoxifillyne (PTF), a tri-substituted xanthine-derived phosphodiesterase inhibitor, has been likely the first agent to be used in studies on cultured human HSC/MFs. PTF resulted in the inhibition of ERK cascade [64], confirming in vivo studies showing its positive anti-fibrogenic activity in an experimental model of liver fibrosis [65]. PTF was found to target ERK activity stimulated by PDGF, then reducing both PDGF-dependent proliferation and chemotaxis in human HSC/MFs, but also to specifically down-regulate TIMP-1 expression favouring degradation of fibrillary collagen [66]. From these initial studies, PTF has been employed by several laboratories as an anti-fibrotic drug and it has also been tested in clinical conditions, with an emerging interest for this agent in the treatment of human NAFLD; in the latter condition, however, PTF action is now being more likely attributed to its ability to target tumour necrosis factor α pathway [67].

#### 5.1.2. *N*-Acetyl Cysteine and Curcumin

Various preclinical studies indicated that in vivo administration of antioxidant molecules could be effective in counteracting fibrogenic progression of chronic liver injury (reviewed in [48,68,69], see later in this review). *N*-acetyl-cysteine was likely the first agent whose effect was shown to result in cell cycle arrest at G1 phase in activated HSC/MFs by modulating the redox state of cysteine residues of Raf-1, MEK, and ERK [70]. These findings provided more specific and MFs-directed basis to employ antioxidant therapies in the treatment of CLD. Similar results have been provided for other antioxidant agents able to target components of ERK cascade in HSC/MFs such as sylibin (an active component of sylimarin) [71] or curcumin [72].

#### 5.1.3. Raf-Kinase Inhibitor Protein

The Raf kinase inhibitor protein (RKIP) is a highly conserved cytosolic peptide that acts, in its non-phosphorylated form, as an inhibitor of Ras/Raf/MEK/ERK signalling pathway. RKIP operates by interacting with the kinase domain of Raf-1 and disrupting Raf/MEK interaction, then preventing the activation of MEK and downstream components [73]. When phosphorylated, RKIP can dissociate from Raf-1 to combine with GRK-2, a negative regulator of G-protein-coupled receptors (GPCRs); phosphorylation of RKIP by PKC stimulates both the Raf/MEK/ERK and the GPCR pathways [74,75]. Interestingly, RKIP expression was down-regulated in activated and proliferating HSC whereas the same cell type was expressing in parallel up-regulation of pRIPK, pRaf, and pERK. Moreover, transfection of activated HSC to overexpress RKIP significantly inhibited phosphorylation of RKIP, Raf, and ERK. Accordingly, the use of locostatin, a pharmacological inhibitor of RKIP, inhibited RKIP expression and significantly reverted phosphorylation of pRIPK, pRaf, and pERK. Overall, RKIP inhibited HSC proliferation by targeting the ERK pathway, although RKIP promoted migration of these cells [76]. These data were confirmed by two studies from the same research group. The first study showed that RIPK down-regulation (obtained by employing locostatin in vivo) resulted in an exacerbation of liver injury and collagen deposition. The second study reported that in vivo administration of didymin, a molecule negatively affecting ERK and PI3/Akt pathway by up-regulating RKIP expression, alleviated liver fibrosis in the rat chronic model of liver fibrosis induced by carbon tetrachloride (CCl4) [77,78].

#### 5.1.4. MAPK Tumour Progression Locus 2

Tumour progression locus 2 (Tpl2, also known as Cot or MAP3K8) is a serine-threonine kinase with an important role in TLR as well as tumour necrosis factor (TNF), IL-1, and G-protein-coupled receptor-mediated signalling [79]. Activation of Tpl2 requires IκB kinase (IKK)-β-catalyzed phosphorylation of the p105 nuclear factor κB (NF-κB) protein, which is complexed with Tpl2 in its inactive state. Following ubiquitination and proteasome-mediated processing of p105 to its shorter p50 form, the complex releases Tpl2 which then becomes catalytically active. The major biochemical function of Tpl2 is the activation of ERK through direct phosphorylation of the ERK kinases MKK1 and MKK2 [80]. Along these lines, an interesting preclinical study has shown that Tpl2 is critical for the activation of ERK signalling in either Kupffer cells and HSCs responding to stimulation of TLR4 and TLR9. Moreover, HSCs lacking Tpl2 were unable to increase the expression of fibrogenic genes like IL-1β and tissue inhibitor of metalloproteinase 1 (TIMP-1). The potential relevance for liver fibrogenesis of Tpl2 was confirmed in Tpl2^−/−^ mice in the two models of liver fibrosis induced by chronic administration of CCl4 or by feeding the methionine-choline-deficient (MCD) diet. In both models, the lack of Tlp2 resulted in a significant reduction in fibrosis as compared to WT mice [81].

#### 5.1.5. Embryonic Stem Cell-Expressed RAS

As previously mentioned, quiescent HSC can undergo activation/trans-differentiation into the classic MF-like phenotype in the presence of chronic liver injury and of a “pro-fibrogenic environment”. A recent study has been designed to investigate the signalling networks of quiescent versus activated HSC, with a focus on expression changes and activity of RAS family GTPases. This study outlined that a particular GTPase, defined as embryonic stem cell-expressed RAS (ERAS, a peculiar member of the family), is specifically highly expressed in quiescent HSC but down-regulated in activated cells. ERAS can maintain quiescence in normal HSCs by targeting AKT via two distinct pathways driven by PI3Kα/δ and mTORC2. In activated HSC, RAS signalling shifts, due to ERAS inactivation, to Raf/MEK/ERK pathways [82], then favours proliferation, growth, and differentiation of activated HSCs as well as survival to apoptosis induction. However, at present, this potentially interesting study has not been followed by in vivo attempts to investigate the role of ERAS in either murine models of liver fibrosis or in human samples from CLD patients.

#### 5.1.6. Direct Inhibition of RAS and ERK

PDGF, as well as other mitogens, can induce activation of ERK and PI3K pathway in HSC/MFs to promote their proliferation and migration. In some studies, direct in vivo inhibition of RAS has been obtained using its antagonist farnesyl-thiosalicylic acid (FTS); this protocol prevented and even reversed rat liver fibrosis through inhibition of HSC proliferation, induction of apoptosis, and MMP activity [83,84]. Alternatively, another study has been designed to inhibit ERK1 by adenovirus mediated small interfering RNA, a procedure that effectively reduced hepatic fibrosis in rats [85]. At present, these strategies have remained at preclinical stage.

#### 5.1.7. SIRT2 Inhibition

In recent years, it has been proposed that acetylation and deacetylation may have a role in liver fibrogenesis and, indeed, the use of histone deacetylase (HDAC) inhibitors in preclinical studies exerted promising anti-fibrotic effects [86]. HDACs are grouped into four classes and two families: the “classical”, and the silent information regulator2 (Sir2)-related protein (sirtuin) families. Along these lines, a recent study has shown that the selective inhibition or sirtuin 2 (SIRT2) is followed by suppression of the expression of critical pro-fibrogenic genes in activated HSCs. Moreover, inhibition of SIRT2 also resulted in the degradation of c-MYC and suppressed the phosphorylation of ERK. In vivo experiments on a preclinical model of liver fibrosis confirmed that SIRT2 deficiency (i.e., in Sirt2^−/−^ mice) resulted in a significant reduction of liver fibrosis. Interestingly, the same study also provided evidence for overexpression of SIRT2, pERK, and c-MYC protein levels in human fibrosis, proposing the existence of a potentially targetable SIRT2/ERK/cMYC pro-fibrogenic axis [87].

### 5.2. Strategies Designed to Target Signalling Pathways and/or ROS Intracellular Generation Upstream to the Activation of Ras/Raf/MEK/ERK Cascade

Several peptide growth factors, through their interaction with cognate receptor(s), can affect and sustain one or more of the phenotypic responses of activated HSC and/or MFs. The “list” includes at least TGFβ1, PDGF, CTGF/CCN2, EGF, FGF, VEGF, and endothelins, as well as Wnt, Hedgehog, and Notch signalling pathways (reviewed in [1,2,3,4,5,6,7,22]). Accordingly, an impressive number of pre-clinical studies have been performed in the last two decades confirming that the strategy to target these interactions (by using genetically manipulated mice, neutralizing antibodies, pharmacological inhibitors, adenoviral vectors, or siRNAs) was indeed effective in vitro as well as in reducing experimental liver fibrosis. Moreover, these strategies have outlined additional aspects of fibrogenic progression of CLDs, increasing the overall knowledge on hepatic MFs as well as on mechanisms and mediators involved. However, once again, only a minority of the data and concepts emerged from these studies have been translated into clinical trials, with very few trials reporting some benefit in CLD patients. In this review, we will intentionally focus the attention on just two of these strategies: a) strategies designed to target PDGF signalling; this is the prototype of fibrogenic signalling, detected in experimental and clinical conditions of progressive CLD, mainly operating by involving activation of Ras/Raf/MEK/ERK cascade in HSC/MFs; b) strategies designed to target production of intracellular ROS by NADPH oxidase isoforms; this happens in response to the interaction of classic pro-fibrogenic peptide ligands (i.e., those already cited in Section 3, most of them operating through ERK pathway, including PDGF, CTGF/CCN2, TGFβ1, FGF, VEGF, Angiotensin II, etc) with their cognate receptors expressed on hepatic MFs. The interested reader may find more details on strategies and drugs used to counteract, with rather similar approaches, other signalling pathways in more exhaustive reviews [1,2,3,4,5,6,7,22,49].

#### 5.2.1. Strategies to Target Platelet-Derived Growth Factor (PDGF) Signalling Pathway

PDGF, particularly PDGF-BB isoform, is the most potent mitogen for HSC/MFs, as well as one of the most potent chemoattractant for these cells; both these effects involve Ras/Raf/MEK/ERK cascade. However, PDGF-dependent and ROS-modulated oriented migration of HSC/MFs also involves JNK isoforms and PI3K pathways [46,47]. In addition, PDGF also exerts a pro-survival and pro-angiogenic role and contributes to activation/trans-differentiation of HSC into HSC/MFs, then favouring also ECM synthesis and deposition [1,2,3,4,5,6,7,22,88]. Although HSCs can express both α- and β-receptor types, only PDGFR-β is up-regulated during HSC activation in vitro and in vivo, mainly following TGFβ1 exposure [89,90]. In addition, only PDGF-BB and PDGF-DD can bind to PDGFR-β, resulting in downstream phosphorylation of ERK1/2 and protein kinase B (Akt/PKB) of the phosphoinositide-3-kinase (PI3K) pathways, leading to significant proliferation of liver MFs [89,90,91]. Moreover, PDGF-BB and -DD can activate PDGFR-α, possibly through PDGFR-α/β heterodimer formation [92]. According to these premises, several strategies have been designed and positively tested in vitro (inhibition of major phenotypic responses) and in vivo in preclinical models (preventing and/or reducing fibrosis). The list includes (see for more details and original references recent exhaustive reviews [5,6,7,88]) the following strategies:

- to target the PDGFR-β, either by using an antisense strategy [93], a dominant–negative soluble PDGFR-β [94], or by using PDGFR tyrosine kinase inhibitors [88]. For the latter option, several RTK inhibitors have been used either in vivo in preclinical studies or in vitro, including: Imatinib mesylate (imatinib, STI571, or Gleevec), an inhibitor of tyrosine kinases active on both PDGFR-β and –α, that can also affect the bcr-abl fusion protein c-kit and Flt3 [95,96]; Sorafenib, a potent inhibitor of VEGF receptor 2 (VEGFR-2), PDGFR-β, and Raf kinases [97]; Nilotinib, a second generation RTK inhibitor, approximately 20 times more potent than imatinib mesylate, able to affect multiple mechanisms, both in vitro and in vivo, including induction of HSC apoptosis, inhibition of PDGF, TGF-β, and other signal pathways, as well as suppression of neo-angiogenesis [98,99,100]. No one of these drugs, effective in preclinical studies, has been translated and/or approved for anti-fibrotic treatment of CLD, although sorafenib is currently employed to treat patients with advanced HCC.
- to block the binding of PDGF ligands to PDGFR by a neutralizing monoclonal PDGF-B antibody (AbyD3263) [101] or by MOR8457 [102], a highly potent and selective PDGF-BB monoclonal neutralizing antibody, both antibodies being particularly efficient in inhibiting PDGF-BB-induced cell proliferation.
- To target PDGFR-β production by PDGFR-β specific siRNA [103,104] delivered into activated HSC by the hydrodynamics-based transfection method, a strategy employed only in pre-clinical studies.
- To use endogenous inhibitors of PDGF signalling (reviewed in reference [88]). Along these lines, a strategy able to reduce liver fibrosis in vivo (preclinical studies) has been designed and tested in order to down-regulate the expression of secreted protein acidic and is rich in cysteine (SPARC), an ECM protein that can represent low-affinity docking sites or reservoirs for the PDGF growth factors [105,106].

#### 5.2.2. Strategies Designed to Target ROS Production by NADPH-Oxidase Isoforms

Oxidative stress, ROS, and other redox-related reactive intermediates are actively involved in progressive CLDs in either experimental or clinical conditions. According to literature data [1,2,3,4,5,6,7,22,68,69,107,108], oxidative stress, particularly ROS and other reactive intermediates can favor CLD progression through several mechanisms, including: a) through the perpetuation of hepatocyte injury and death, then also resulting in perpetuation of hepatic inflammatory response; b) by directly acting on hepatic MFs or their precursor cells by up-regulating critical profibrogenic genes (including procollagen type I, CCL2, TIMP1, and others) and/or by modulating phenotypic responses by activating specific signal transduction pathways and transcription factors; c) following generation of intracellular ROS by NADPH-oxidase isoforms in hepatic MFs in response to ligand-receptor interaction when these cells are exposed to profibrogenic polypeptide ligands (PDGF, CTGF/CCN2, TGFβ1, FGF, VEGF, Angiotensin II, and others). The latter NADPH-oxidase-dependent option is of particular relevance since it contributes to a persistent shift towards higher intracellular ROS levels in hepatic MFs, an event that is believed to concur in the perpetuation and further amplification of major signaling pathways in these activated cells. Along these lines, NADPH-oxidase (NOX) is a multicomponent transmembrane complex, found in either phagocytic and non-phagocytic cells. This complex generates, in response to numerous stimuli (cytokines, growth factors, adipokines, etc), ROS-like superoxide anion or hydrogen peroxide from molecular oxygen by using NADPH as an electron donor [109]. The NOX present in professional phagocytic cells (i.e., neutrophils and macrophages) is formed by the heterodimeric and membrane bound flavocytochrome b558 complex (containing gp91^phox^ or NOX2 and the regulatory subunit p22^phox^) and by a number of regulatory cytosolic components (Rac, p47^phox^, p67^phox^, and p40^phox^). In the presence of agonists like endotoxin or interferon-γ, the cytosolic components translocate to the membrane to bind the flavocytochrome, an event that is followed by activation of the complex and ROS generation. Similarly, in non-phagocytic cells, NOX2 is replaced by a different member of the NOX family (including NOX1, NOX3, NOX4, NOX5, DUOX1, and DUOX2). Hepatic MFs, in particular those derived from activated HSCs, express NOX2 as well as NOX1 and NOX4; following exposure to a long list of peptide ligands (including LPS, Angiotensin II, TNF, and interferon-γ, but also PDGF, EGF, bFGF, and endothelins) the complex is formed and activated [5,6,7,107,108]. ROS generated in this way can affect MAPK cascades (including ERK pathway), as well as PI3K/Akt signaling and NF-kB activation, then sustaining the major phenotypic responses (proliferation, ECM synthesis, migration, pro-inflammatory, etc.). All these considerations led to using specifically designed pharmacological NOX inhibitors in order to specifically target NOX-mediated and ROS-dependent phenotypic responses. This was an interesting approach to circumvent the essential lack of antifibrotic efficacy in clinical trials of generic antioxidants (vitamin E, vitamin C, sylimarin, or others) and at the same time to target molecules like NOX that are activated by multiple peptide mediators [1,2,3,4,5,6,7,22,68,69,107,108]. At present, a specific dual NOX4/NOX1 inhibitor, GKT137831, has been tested in different pre-clinical murine models of liver fibrosis and found to be able to significantly prevent ECM deposition (i.e., likely by targeting MFs) and to counteract inflammatory response as well as other ROS-related events (cell death/apoptosis) [110,111,112]. This approach is then potentially promising, keeping in mind that NOX4 has been found to be up-regulated in human fibrotic liver specimens.

## 6. Summary

Extracellular signal-regulated kinase (ERK) signaling pathway is a critical one in modulating major phenotypic responses of liver myofibroblasts. These critical pro-fibrogenic cells, whatever their origin and the etiology of the specific CLD, are believed to significantly contribute to CLD progression towards advanced fibrosis, leading eventually to cirrhosis and related complications, as well as to liver failure or development of hepatocellular carcinoma. According to pre-clinical studies and clinical studies and trials performed in the last two decades, we have presented in this review the major roles played by ERK signaling pathways in mediating the phenotypic responses of liver MFS, as elicited by a number of extra- and intracellular signals or mediators. We also offered a selection of the major strategies and drugs employed in the never-ending attempt to elaborate efficient anti-fibrotic therapies with a specific focus on those designed to directly or indirectly interfere with ERK signaling to then negatively affect MF-dependent pro-fibrogenic responses. 

## Abbreviations

α1-AT	α1-anti-trypsin
AhR	Aryl hydrocarbon receptor
AIH	Autoimmune hepatitis
ALD	Alcoholic liver disease
bFGF	Basic fibroblast growth factor
CCL2	C-C Chemokine motif chemokine ligand 2
CCl4	Carbon tetrachloride
CCNCyr61	Cysteine-rich protein 61
CLD	Chronic liver disease
DAA	Direct antiviral agents
DOX	Dual oxidase
ECM	Extracellular matrix
EGF	Epidermal growth factor
EMT	Epithelial mesenchymal transition
ERAS	Embryonic stem cell-expressed RAS
ERK	Extracellular signal-regulated kinase
ET-1	Endothelin-1
FTS	Farnesylthiosalicylic acid
FXR	Farnesoid X receptor
GATA4	GATA binding protein 4
GIV	Gα-interacting vesicle-associated protein
GPCRs	G-protein-coupled receptors
GRB2	Growth factor receptor-bound protein 2
HCB	Hepatitis virus C
HCC	Hepatocellular carcinoma
HCV	Hepatitis virus C
HDAC	Histone deacetylase
HNE	4-hydroxy-nonenal
HPC	Hepatic progenitor cells
HSC	Hepatic stellate cells
IKK	IKB kinase
KC	Kupffer cell
KLF6/KLF2	Kruppel-like factors
LXR	Liver X receptor
MAPK	Mitogen-activated protein kinase
MCD	Methionine-choline-deficient
MFs	Myofibroblasts
MMPs	Metalloproteinases
MMT	Mesothelial mesenchymal transition
MRTF-A	Myocardin-related transcription factor A
NAFLD	Non-alcoholic fatty liver disease
NF-κB	Nuclear factor κB
NOX	NADPH-oxidase
NR4A1	Nuclear receptor subfamily 4 group A member 1
PARP1	PolyADP ribose-polymerase 1
PBC	Primary biliary cholangitis
PDGF	Plateled-derived growth factor
PI3K	Phosphoinositide-3-kinase
PKB	Protein kinase B
PKC	Protein kinase C
PSC	Primary sclerosing cholangitis
PTF	Pentoxifillyne
REV-ERBα	Nuclear receptor subfamily 1 group D member 1
RKIP	Raf kinase inhibitor protein
ROS	Reactive oxygen species
SEC	Sinusoidal endothelial cells
Sir2	Silent information regulator 2
SIRT2	Sirtuin 2
SOS	Son of Sevenless
SOX9	Sex-determining region Y-box 9
TGFα	Transforming growth factor α
TGFβ1	Transforming growth factor- β1
TIMPs	Tissue inhibitor of MMPs
TLRs	Toll-like receptors
TNF	Tumor necrosis factor
Tpl2	Tumor progression locus 2
VDR	Vitamin D receptor
VEGF-A	Vascular endothelial growth factor A
VEGFR-2	VEGF receptor- 2
WD	Wilson’s disease
YAP	Yes associated protein
